# Measurement of Bone Mineral Density in Children with Cerebral Palsy from an Ethical Issue to a Diagnostic Necessity

**DOI:** 10.1155/2020/7282946

**Published:** 2020-09-19

**Authors:** Jasmin S. Nurković, Pavle Petković, Danijela Tiosavljević, Radiša Vojinović

**Affiliations:** ^1^Center for Regeneration and Rehabilitation, Novi Pazar, Serbia; ^2^Radiology Department, Faculty of Medical Sciences, University of Kragujevac, Serbia; ^3^Radiology Department, Clinical Center Kragujevac, Serbia; ^4^Department of Humanities, Medical Faculty, University of Belgrade, Serbia

## Abstract

**Introduction:**

Due to concerns about cumulative radiation exposure in the pediatric population, it is not standard practice to perform dual-energy X-ray absorptiometry (DXA) analysis in the diagnostic process of musculoskeletal disorders, such as cerebral palsy (CP). This study aimed to evaluate the bone mineral density (BMD) in children with CP and the ethical justification of applying DXA analysis in these children. *Material and Methods*. In this monocentric retrospective analysis, data were collected from children and adolescents with CP who were treated for a primary illness for three years. A clinical examination, which included a DXA analysis, recommended by the multidisciplinary team, was performed. After applying inclusion and exclusion criteria, 60 scans remained for statistical analysis. BMD and *Z*-scores for the lumbar spine (LS), and hip right and left femoral neck (RFN and LFN, respectively), and total hip (TH) were recorded.

**Results:**

The average age of children with CP when DXA analysis was first performed was about 7 years. The BMD (mean ± SD) at LS (LS-BMD) of all patients was 0.612 ± 0.12, at RFN 0.555 ± 0.11, at LFN 0.572 ± 0.1, and at TH (TH-BMD) 0.581 ± 0.13. The values of the *Z*-score (mean ± SD) at LS of all patients were −2.5 ± 0.22, at RFN −2.2 ± 0.21, at LFN -2.25 (SD = 0.2), and at TH -2.3 (SD = 0.23). There was no statistical significance between age and gender; however, BMI, walking ability, fracture history, and pattern of CP had a significant impact on BMD and *Z*-score values of these children.

**Conclusion:**

The results of our study clearly indicate that children with CP have a higher risk of low BMD, osteoporosis, and bone fractures, which makes it ethically justifiable to perform the DXA analysis in these children.

## 1. Introduction

The current standard for measuring bone mineral density (BMD) is dual-energy X-ray absorptiometry (DXA), due to its availability, accuracy, ease of repetition, and patients' low X-ray exposure [1]. Using DXA analysis in pediatric population is complex, because of the bone mineral density changes during the process of growth and development. Children have not achieved their final bone mineral density; thus, every obtained result needs to be compared to the average value for the same sex and age (*Z*-score), which means that the results vary at different ages, unlike adults, whose measured BMD value is compared to one standard, an “ideal” value for a grown, mature person (*T* score) [2]. Children of the same age can differ in skeletal maturity, which further adds complexity to the interpretation of DXA results. In children who have musculoskeletal disorders, e.g., cerebral palsy (CP), DXA analysis becomes even more challenging [3, 4].

CP is a term used for describing abnormal motor development of central origin, and it is the most prominent neurological disorder that occurs in childhood and continues into adulthood [5]. In developed countries, the incidence of this disorder is about 2 per 1000 live births. 70-80% of cases have a prenatal, unknown cause, while a smaller percentage occurs due to labor complications and problems in the early neonatal period [6]. CP patients have lower life expectancy compared to the general population, especially when severe complications and comorbidities are present [7]. Children with CP face numerous complications during their development: gastroesophageal reflux, aspiration syndromes, respiratory infections, seizures, and muscle contractures. In addition, aging-associated diseases like atherosclerosis, osteoporosis, sarcopenia, osteoarthritis, and dementia are more pronounced and occur earlier in CP patients, due to their static lifestyle. It is known that bone fractures are not a direct cause of death; however, their influence on morbidity is high [8]; therefore, measuring BMD is an important diagnostic procedure [9, 10]. For low BMD-related fracture risk, osteoporosis assessment, and therapeutic possibilities are less defined, compared to adult population. However, in the last years, there has been progress regarding diagnostics and diagnostic classification of reduced BMD in children. International Society of Clinical Densitometry (ISCD) defined parameters for osteoporosis in children in 2008 [11].

In children, osteoporosis diagnosis is based on two criteria: (1) the presence of low BMD with a *Z*-score lower than -2.0 and (2) presence of fracture history (long bones of the upper and lower extremities, compression fractures of the vertebrae) [11, 12]. Certain researchers consider that the term “osteopenia” should be avoided in children and that referring to *Z*-score results between 0 and -2 as “low BMD” is more appropriate [11]. Risk factors for low BMD values in children with CP are low weight, anticonvulsive therapy, poor nutrition, and inadequate sun exposure [10–12]. The incidence of bone fractures in children with a severe form of CP is 7-9.7% per year, with fractures of the lower extremity of the femur being the most common.

Every pediatric X-ray exam, especially in those with psychomotor development impairments, represents an ethical dilemma, and the benefit of a diagnostic or therapeutic procedure must outweigh the risks of the procedure itself [13]. Applying the principles of informed consent in pediatric healthcare involves consent by the parent/guardian, as well as the consent of a child older than 15. Consent should be the result of a continuous interactive communication between the health care providers, the child, and the parent/guardian. In the process of obtaining consent, health care providers are obliged to include the child in decision-making, according to its maturity and cognitive abilities, and provide the child, as well as the parent/guardian, with a form and amount of information that would be comprehensive and clear. Older children and adolescents, without cognitive deficits, have a right to independently consent to a suggested medical procedure, provided they are adequately informed about all the aspects of it by the health care provider. In the case of them declining the medical procedure, the health care provider is obliged to ask the patient's legal guardian for consent [13].

In case of DXA analysis, for spinal and femoral scanning, the effective radiation dose varies between 0.5 *μ*Sv and 15 *μ*S for adults, while, in children, these values are higher and can reach 20 *μ*S [14]. By comparison, the average effective dose acquired by each individual from natural sources is about 10 *μ*S. The usual duration of a scan for cooperative children is about 1 minute for the lumbar spine and 5-7 minutes for the whole body [10, 14].

Numerous studies have shown that physical exercise can improve bone metabolism, increase BMD and BMC, provide good bone structure and strengthen bone mass [15–17]. Therefore, physical and kinesitherapy treatment, which is an integral part of the treatment of these children, is very important, both because of the stimulation of motor development and the improvement of BMD.

This study aimed to evaluate the BMD in children with CP and ethical justification of applying DXA analysis in these children. Our hypothesis was that the obtained data would show significantly low values of BMD and *Z*-score, which increases the risk of bone fractures and that it would highlight the need for an adequate treatment, which is aimed at damage prevention. Our results may favor the need for exercise and kinesiotherapy in children with CP.

## 2. Material and Methods

### 2.1. Study Design and Population

The present study is a monocentric retrospective analysis. Data were collected from children and adolescents with CP who were treated for a primary illness in a Center for Regeneration and Rehabilitation at Novi Pazar, Serbia, during a three-year period, from March 2017 to March 2020. A detailed clinical and radiological examination, which included a DXA analysis, recommended by the multidisciplinary team, physiatrist, child neurologist, radiologist and pediatrician, was carried out prior to the start of physical treatment in these children. This study was approved by our local institutional review and ethical board. All patients/caregivers provided a written informed consent to participate. All of the investigations have been conducted according to the ethical principles suggested in the Declaration of Helsinki. Measures have been made to protect the privacy of research subjects and the confidentiality of their personal information.

**Figure 1:**
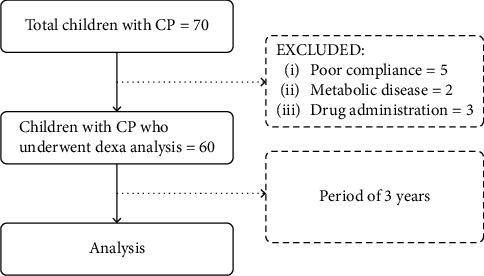
Participant flow diagram.

During the course of three years, 70 children with CP were treated in our Center. 65 DXA scans were performed. In 5 children, the analysis could not be done due to poor compliance. The inclusion criteria were diagnosis of CP, Caucasian ethnicity, age 3 to 18 years, and written informed consent to full medical examination from patients, when possible, or from their parents/caregivers. The exclusion criteria were diseases primarily involving bone metabolism or familial history of bone metabolism disorders; chronic diseases with stunted growth, e.g., renal failure; and corticosteroid or growth hormone medication. The exclusion criteria were evaluated on the basis of patient records, due to the retrospective design of the study; this represents an important limitation of our study. After applying inclusion and exclusion criteria, 60 scans remained for statistical analysis. In 2 cases, kidney disease was the reason for exclusion from this study, 3 patients had previously received corticosteroid or growth hormone medication (Figure 1).

The CP patterns were classified as hemiplegic, diplegia, triplegia, or quadriplegic, in accordance with the criteria of the Surveillance of Cerebral Palsy in Europe [18].

### 2.2. Anthropometry

In children who were able to stand, body length was assessed with a stadiometer (SECA®, model No. 213, Hamburg, Germany) in 0.1 cm increments. In children who were unable to stand, a retractable metal tape was used. The body weight was measured by a digital scale in 0.1 kg increments. Body mass index (BMI) was calculated as a person's weight in kilograms divided by the square of their height in meters (kg/m^2^).

### 2.3. DXA Measurement and Data Acquisition

DXA scans were obtained using a HOLOGIC densitometer, model Discovery Ci, by a single medical technician. Subjects' DXA scans over a 3-year period were utilized, as this corresponded to the time that studies at our institution were performed on a single machine, modeled on the study of our colleagues from Germany and USA [19, 20]. The lumbar spine and both hips were imaged, when possible, and analyzed with the manufacturer's software. A daily calibration of the machine was done with the spine and whole body phantom and weekly calibration with the tissue bar and air scan; DXA precision determination with current staff demonstrates a coefficient of variation of ≤1.0% at the spine and ≤2.0% at the femoral neck.

**Figure 2:**
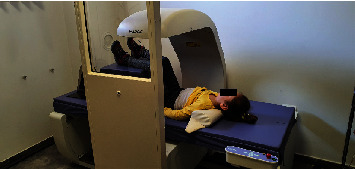
Positioning on the device and clothing of the patient was executed according to the manufacturer's instructions. Photographing and publishing a photograph of a child in this scientific paper is approved by the parents. The principles of the United Nations “Convention on the Rights of the Child” (UNCRC) and “Reporting guidelines to protect at-risk children” were respected. The rights to the photo are reserved by the authors.

Quality control assurance measurements were performed according to the manufacturer's recommendations. Positioning on the device and clothing of the patients were executed according to the manufacturer's instructions (Figure 2). Only measurements that were considered evaluable, e.g., without movement artifacts, were included in the analysis. BMD and *Z*-scores for the lumbar spine (LS), and right and left femoral neck (RFN and LFN, respectively), and total hip (TH) were recorded.

### 2.4. Statistical Analyses

Descriptive statistics were obtained, and the Shapiro-Wilk test was used to evaluate the normal distribution of the data. Subjects' demographic, functional, and bone density characteristics were summarized by mean (SD) for continuous variables, frequency (percentage) for nominal variables, and rates for percentage change. For group comparison, two-sample *t*-test was used for continuous variables. For gender, differences in BMD and *Z*-scores between girls and boys were evaluated. One-way ANOVA analysis with Bonferroni correction of variance was used to evaluate if there is a significant difference in BMD or *Z*-scores among the BMI groups (underweight vs. health weight vs. overweight vs. obese) and walking ability groups. Morever, a comparison between quadriplegic and other patterns of CP was performed. Changes in BMD and *Z*-scores was calculated only in subjects who did not receive pharmacologic treatment (beyond optimization of vitamin D and calcium intake).

## 3. Results

Demographic and medical information is presented in Table 1. The average age of children with CP when DXA analysis was first performed was about 7 years. The youngest patient was 3, and the oldest was 17 years old. Puberty, as a crucial period in the growth and maturation of children, was used to classify the children in prepubertal and pubertal groups, which were then compared by their BMD and *Z*-score values. The age of 11 years was taken as the limit [21], and there were 42 prepubertal children and 18 older than 11 years. Of the total number of children examined, there were 28 boys (46.67%) and 32 girls (53.33%). When it comes to anthropometric measures, the average body weight of children was about 25 kg, height (or length in immobile patients) 125 cm, and BMI in 51.67% of patients was normal. Overweight (85^th^-95^th^ percentile) was present in 23.33% of the children tested, with several extreme cases, 12 underweight children (less than 5^th^ percentile) and 3 children with obesity (greater than 95^th^ percentile).

Walking ability is an important factor for proper development of the musculoskeletal system. Unfortunately, as many as 68.33% of the surveyed children did not walk, 16.67% of the children had mobility with the help of various aids, and only 15% of the respondents walked independently. There were 14 (23.33%) children with a history of fracture, and 46 (76.67%) had no bone fracture before or during the study (Table 1).

**Figure 3:**
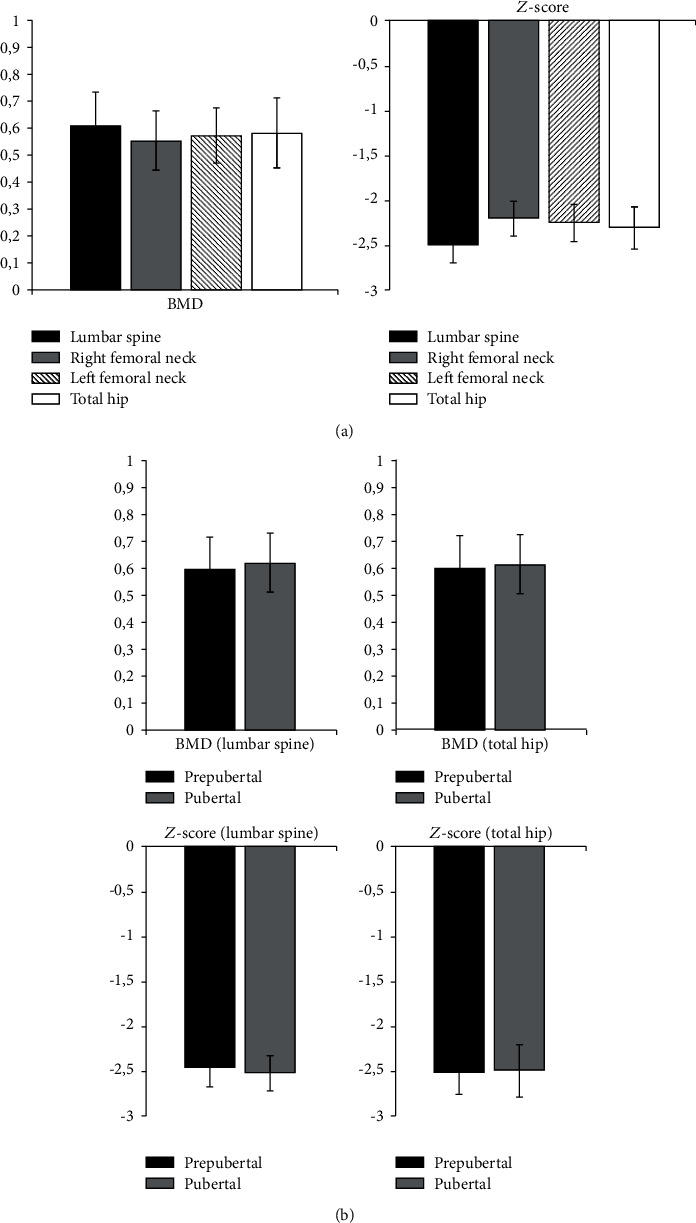
BMD (g/cm^2^) and *Z*-score of all patients (a) and in relation to puberty (b).

In our study, the BMD (mean ± SD) at LS (LS-BMD) of all patients was 0.612 ± 0.12, at RFN 0.555 ± 0.11, at LFN 0.572 ± 0.1, and at TH (TH-BMD) 0.581 ± 0.13. The values of the *Z*-score (mean ± SD) at LS of all patients were −2.5 ± 0.22, at RFN −2.2 ± 0.21, at LFN -2.25 (SD = 0.2), and at TH -2.3 (SD = 0.23) (Figure 3(a)). These values did not significantly deviate from previous literature data ^(8, 10, 15)^. A comparison of the obtained values at LS and TH regions with respect to puberty is shown in Figure 3(b). It can be seen that there was no statistically significant difference between children in prepubertal and pubertal period (Figure 3(b)).

**Figure 4:**
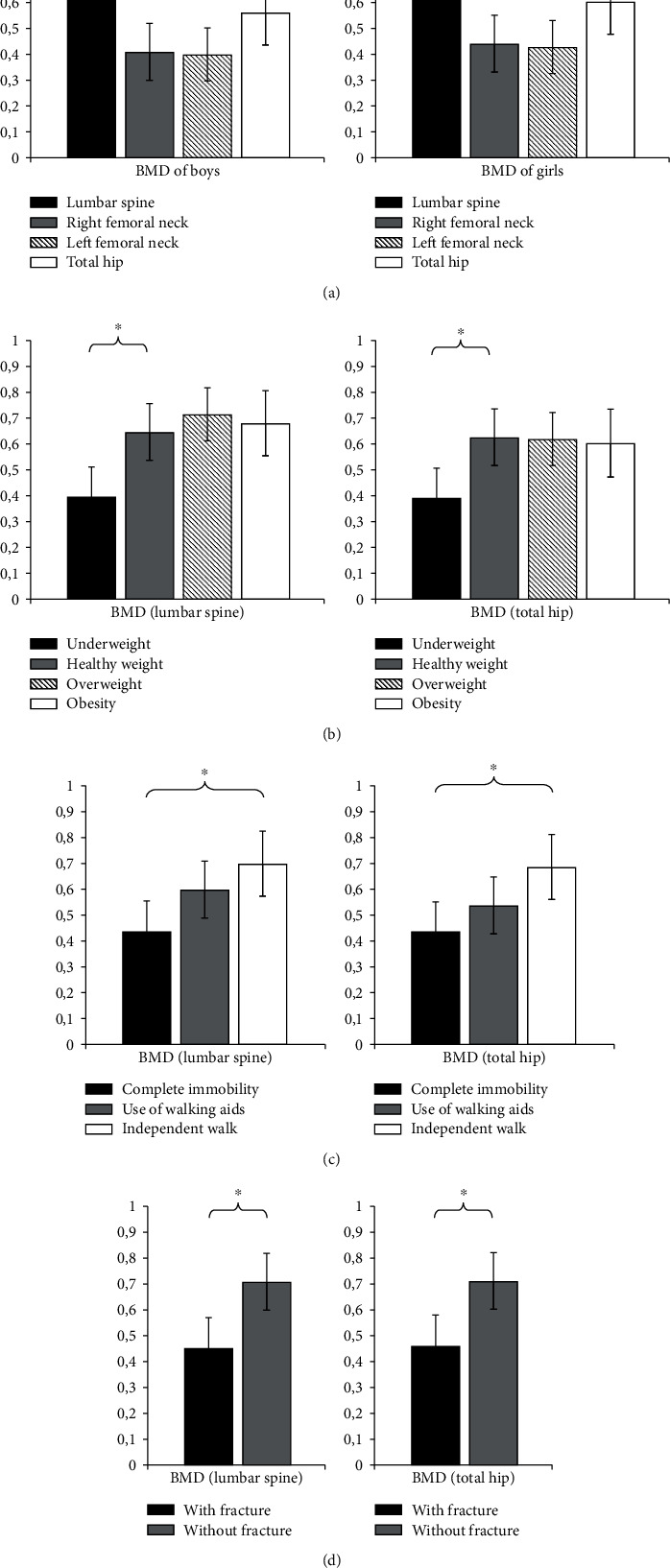
BMD (g/cm^2^) of children with CP according to patient characteristics: (a) sex, (b) weight, (c) walking ability, and (d) fracture history; ^∗^*p* < 0.05.

Likewise, a comparison of BMD with respect to gender was done. There was also no statistical significance between boys and girls (Figure 4(a)). In underweight children, LS-BMD (0.388 ± 0.12) was significantly lower than that in children with healthy, overweight, and obesity values (*p* < 0.05) (Figure 4(b)). Similar values were observed for the TH region. Complete immobility has proven to be an important parameter for BMD values. Children who were not mobile had significantly lower LS-BMD (0.433 ± 0.12) and TH-BMD (0.431 ± 0.12) than children who used aids or were walking independently (*p* < 0.05) (Figure 4(c)). Statistical significance between the LS-BMD and TH-BMD values in children who had fractures (0.45 ± 0.12 and 0.46 ± 0.12, respectively) and those without fractures (0.71 ± 0.11) was expected and is shown in Figure 4(d) (*p* < 0.05).

**Figure 5:**
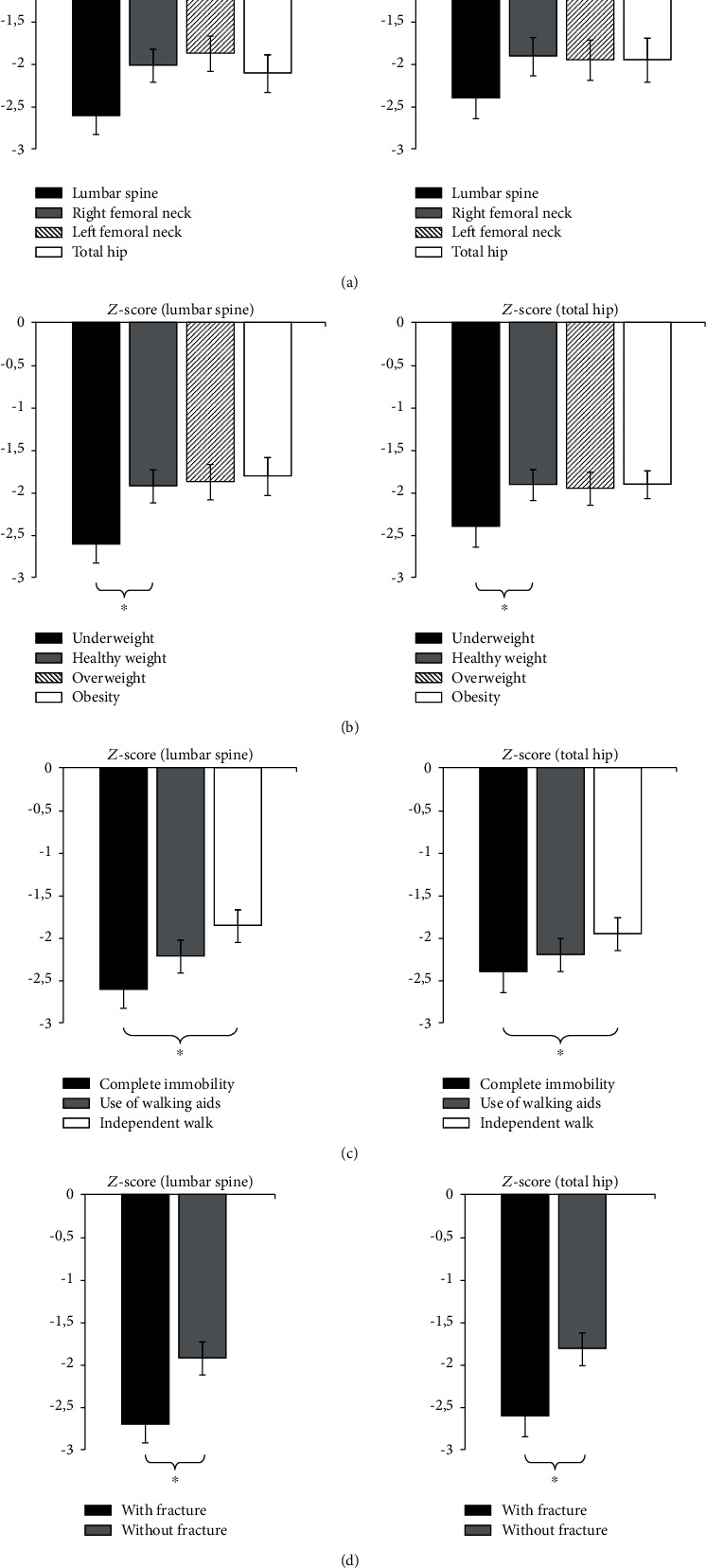
*Z*-score of children with CP according to patient characteristics: (a) sex, (b) weight, (c) walking ability, and (d) fracture history; ^∗^*p* < 0.05.

Analyzing the obtained values of *Z*-score against the same parameters, there was also statistical significance in patients with reduced BMI and immobility, as with those who have had fractures in the past. There was no statistically significant difference in *Z*-scores between boys and girls (Figure 5(a)–5(d)).

**Figure 6:**
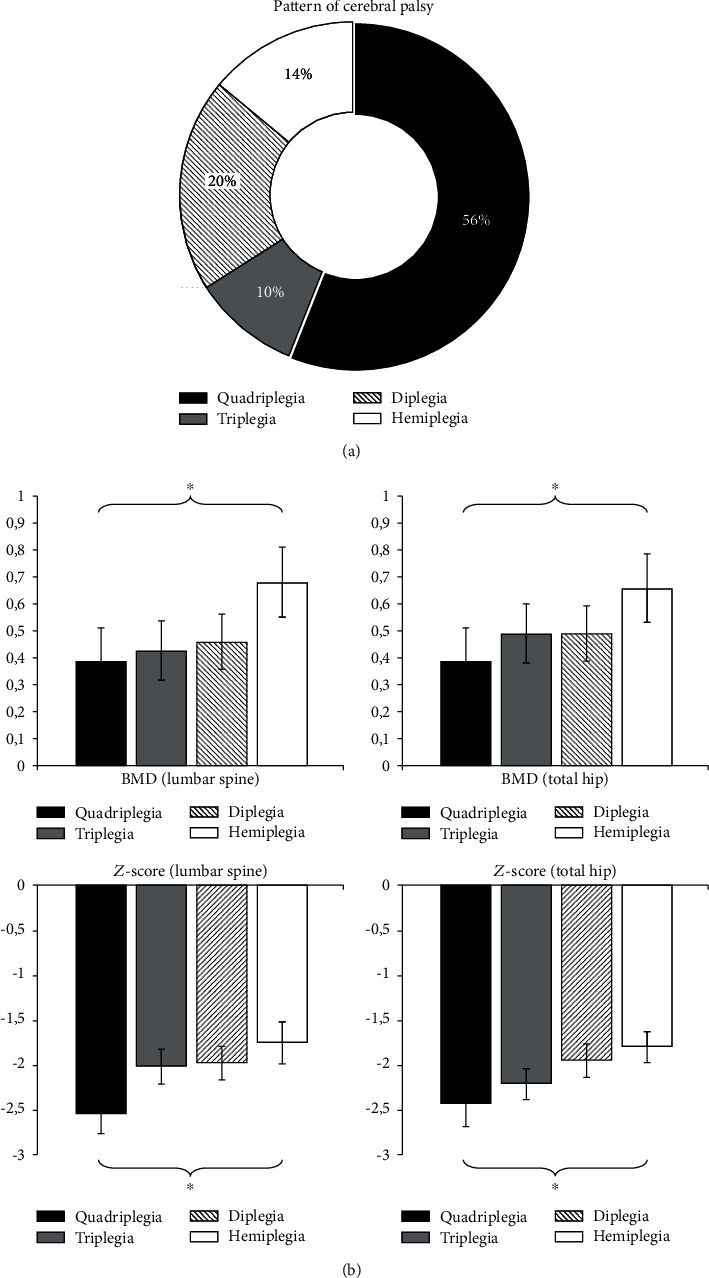
Percent of pattern of CP (a), and BMD (g/cm^2^) and *Z*-score of all patients and in relation to pattern of CP (b); ^∗^*p* < 0.05.

Finally, we compared values of BMD and *Z*-score in patients who had different clinical patterns of CP.  Figure 6(a) shows that most were children with quadriplegia, as much as 56%. Diplegia was present in 20% of cases, hemiplegia was 14%, and triplegia was only 10%. Statistical analysis showed that the BMD and *Z*-score values were lower in children with quadriplegia (LS-BMD 0.388 ± 0.12; TH-BMD 0.389 ± 0.12; and LS-Z-score −2.55 ± 0.22; TH-Z-score −2.45 ± 0.25), compared to children with hemiplegia (*p* < 0.05), and there was no difference in comparison with triplegia and diplegia (Figure 6(b)).

## 4. Discussion

Reduction in BMD was first reported in 1994, in nine non-ambulant patients with CP [22]. Several further studies have shown low BMD values in children with CP, with results ranging from 68% to 97% of the examined children [23–27]. In our study, based on the latest recommended diagnostic criteria [23, 26, 27], all of the examined children had osteopenia, i.e., reduced BMD, and 23.33% of them had osteoporosis. A percentage this high could be explained, by the families of the patients living in less-developed areas of our country, and that the majority of patients grew in poor conditions, which had a significant impact on nutrition and care of the children [28, 29].

In childhood and adolescence, around 50% of the complete adult bone mass is formed [30]. In a 2015 Chinese child and adolescent health study, insufficient bone mineral content (BMC) is a result of insufficient physical activity and inadequate nutrition [31]. Calcium, as the main component of BMC, is one of the most-researched nutrients related to bone health [32]. Children with CP have various difficulties breastfeeding, swallowing, and/or chewing, which reflect itself on quality and adequate food intake and BMI. Alternative feeding methods are often used: nasogastric tube or gastrostomy [33]. Our results have shown that 20% of children had a reduced body mass and total BMI (below 5^th^ percentile for - underweight), which significantly affected the BMD and our results.

Increasing evidence shows that BMD is related to muscle function in healthy children [16]. Physical activity is of extreme importance when it comes to bone mass accumulation in children and adolescents, and it is observed that, in a certain degree, physical exercise can compensate for the lack of calcium intake [34]. Regular medium to high-intensity physical activity has a positive impact on bone health in young population [35, 36]. In a recent American Physical Activity Guidelines Advisory Committee Report, it is recommended that children and adolescents, ages 6-17 years, have 60 minutes or more of medium to high-intensity physical activity on a daily basis [37]. The 2016 Chinese physical activity guideline for children recommends at least 60 minutes of medium-high intensity physical activity per day, as well [38]. It is noted that the degree of physical activity is in direct correlation with BMD level [27, 34], which our study confirms. Children with quadriplegia had very low values of BMD and *Z*-score, and there was a statistical significance even when compared to children with only a certain degree of motor skills. Chen et al. [16] compared the results of two studies in which the results indicate that high-level physical activity with antigravity muscle training under safety control (such as jumping under supervising and necessary support), probably produces greater bone strains (≥1,500–2,500 microstrains) or eight times the body weight in ambulatory children with CP [39, 40]. Our finding supports the hypothesis that lower motor capacity and lesser mobility existing in this group of pediatric patients can affect BMD independently of the other risk factors. In addition, weakened muscle function and lack of mechanical load, present in our patients, result in inadequate forming and modeling of the muscle tissue, which consequently leads to reduced muscular strength and abnormal bone geometry and mineral content [41]. The meta-analysis published in late 2017 shows that weight-bearing exercise significantly improved the BMD in the femur compared with pretreatment values but had no effect on the BMD of the lumbar spine [42]. Mechanical stress on bone is a determinant of bone morphology, BMD, and bone strength [42, 43]. In children with CP who have difficulties in maintaining an upright position, less mechanical force is transmitted to the femurs than the lumbar spine, because mechanical stress can be imposed on the lumbar spine in a seated position.

Low BMD is asymptomatic by itself. However, fractures that can occur cause pain and significantly contribute to the comorbidity present in this pediatric population [8]. The most common location for these is the diaphysis of long bones, e.g., the distal extremity of the femur. In our study, 14 children had fractures in their medical history, which classified them as children with osteoporosis. Considering the existing issues regarding insufficient drug efficiency and difficulties in establishing optimal dosage, as well as potential adverse reactions to drugs, pathological fractures add even more complexity to the already challenging treatment course [44].

In our study population, we found no correlation between the gender and age of children with low BMD results (Figures 3–5). On the contrary, in previous studies, it was often reported that the sex and age (prepubertal and pubertal period) were correlated with low BMD in children with CP [45, 46], most probably due to differences in sex hormone levels. We have no other explanation for these characteristics having no impact on BMD in our study population, except that the hormone effect (at this age) probably had not yet begun.

Taking into account the abovementioned facts, as well as the results of our study, it is clear that children with CP have a higher risk of low BMD, osteoporosis, and bone fractures. Children with CP require radiologic diagnostic procedures more often, due to the need to monitor their hip migration in order to prevent dislocation, frequent injury, and bone/joint deformities. However, exposing them to additional X-radiation during nonstandard procedures, such as DXA analysis, represents an ethical question. Exposure to X-radiation carries a greater risk of malignancy, especially leukemia and thyroid cancer, in children than in adult population [47]. Previous studies have shown that many healthcare providers, including clinicians, even radiologists, are unaware of potential radiation-related risks in case of excessive and unnecessary diagnostic tests [48]. In practice, the patients often undergo tests before or without consenting to a procedure. In addition, alternative diagnostic procedures intentionally remain undiscussed. The fact that it takes the effects of the procedures 5 to 20 years to manifest them adds even more complexity to the issue [49]. Medical situations most frequently provoke multiple medical and ethical principles simultaneously and require moral and ethical judgment and commitment. Given that this study adequately and in line with the legislation of the Republic of Serbia [47] applies the ethical concept of informed consent in the pediatric patient population, the medical situation involving the diagnostic application of DXA analysis in this group of pediatric patients is ethically analysed here through the double effect phenomenon, due to the above highlighted concerns about cumulative radiation exposure in the pediatric population. The “double effect” of syntagm in medical ethics describes those practices of medical professionals that are known to have, in addition to the positive, simultaneously or successively negative consequences [50]. Led by the results of this study, which open the door to great and significant therapeutic opportunities in the field of practical prevention in this patient population, we can conclude that the benefit of this diagnostic procedure outweighs the risks of the procedure itself.

According to that, the use of DXA analysis in the pediatric population of patients with CP for the purpose of determining BMD is considered as one of those medical situations where the ethical principle of beneficence (*Salus aegroti suprema lex*—well-being of the patient is the supreme law) becomes more binding than the principle of no maleficence (*Primum non nocere*—do no harm), which were simultaneously provoked.

Its rational application in this patient population, as a positive balance of benefits and harms from it, opens the possibilities for further significant action of medical professionals who achieve the best final results for their health. It is an active action of medicine towards the prevention and reduction of the risk of further damage to health, the onset of pain and suffering, a significant deterioration in the quality of life and an additional shortening of the life expectancy of vulnerable pediatric patients with CP. Therefore, according to the result of our study, we strongly recommend this diagnostic procedure in the clinical monitoring of children with CP.

There are several limitations to our study. As in similar studies of this kind [19], the present study is a retrospective analysis of data obtained as part of a standard clinical care during an intensive rehabilitation treatment. There may be selection bias because the sample of all children with CP is not representative. In addition, anamnestic or heteroanamnestic fracture data could not be fully verified. Lower limb contractures and deformities, a common finding in children with CP, can affect the height measurement and thus the BMI calculation in the study.

## 5. Conclusion

The present study has confirmed that CP may worsen bone health in children and adolescents. BMD and *Z*-score values in children with CP were significantly lower than the reference limits. Furthermore, BMI, walking ability, fracture history, and pattern of CP had a significant impact on BMD and *Z*-score values of these children. All this confirms our hypothesis that CP increases the risk of bone fractures and that timely diagnosis and ensuring regular and persistent kinesiotherapy would be necessary in this pediatric population. Therefore, the use of DXA analysis outweighs the potential detrimental effect of X-radiation and we strongly recommend it in the clinical monitoring of children with CP.

## Figures and Tables

**Table 1:** Participants characteristics.

	Total (*N* = 60)
Age at first DXA, years *M* (range)	7.6 (3.9–10.1)
≤11 years—prepubertal, *N* = 42 (SD)	5.2 (2.4)
>11 years—pubertal, *N* = 18 (SD)	13.8 (4.2)
Sex	
Boys, *N* (%)	28 (46.67)
Girls, *N* (%)	32 (53.33)
Anthropometrics	
Weight (kg), *M* (SD)	25.4 (8.1)
Height (cm), *M* (SD)	125 (44)
BMI (kg/m^2^), *M* (SD)	16.6 (5.6)
Underweight (less than 5^th^ percentile), *N* (%)	12 (20)
Healthy weight (5^th^-85^th^ percentile), *N* (%)	31 (51.67)
Overweight (85^th^-95^th^ percentile), *N* (%)	14 (23.33)
Obesity (greater than 95^th^ percentile), *N* (%)	3 (5)
Walking ability	
Complete immobility, *N* (%)	41 (68.33)
Use of walking aids, *N* (%)	10 (16.67)
Independent walk, *N* (%)	9 (15)
Fracture history	
Yes, *N* (%)	14 (23.33)
No, *N* (%)	46 (76.67)

*M*: mean; *N*: number; SD: standard deviation; BMI: body mass index.

## Data Availability

The data that support the findings of this study are available on request from the corresponding author. The data are not publicly available due to restrictions, their containing information that could compromise the privacy of research participants.
